# Draft Genome Sequence of Anaplasma marginale Strain Mex-01-001-01, a Mexican Strain That Causes Bovine Anaplasmosis

**DOI:** 10.1128/MRA.01101-18

**Published:** 2018-10-25

**Authors:** Rosa Estela Quiroz Castañeda, Itzel Amaro-Estrada, Fernando Martínez-Ocampo, Sergio Rodríguez-Camarillo, Edgar Dantán González, Mayra Cobaxin-Cárdenas, Jesús Francisco Preciado-de la Torre

**Affiliations:** a Unidad de Anaplasmosis, Centro Nacional de Investigación Disciplinaria en Parasitología Veterinaria, INIFAP, Carretera Federal Cuernavaca-Cuautla No. 8534, Jiutepec, Morelos, Mexico; b Laboratorio de Estudios Ecogenómicos, Centro de Investigación en Biotecnología, Universidad Autónoma del Estado de Morelos, Cuernavaca, Morelos, Mexico; Louisiana State University

## Abstract

Bovine anaplasmosis is an arthropod-borne hemolytic disease caused by Anaplasma marginale. While only a few Anaplasma marginale strains have been reported, no Mexican strains have been reported. Due to the genetic diversity of A. marginale, the genome of the strain Mex-01-001-01, isolated in Mexico, represents a new source of information.

## Abstract (precis)

Bovine anaplasmosis is an arthropod-borne hemolytic disease caused by Anaplasma marginale. While only a few Anaplasma marginale strains have been reported, no Mexican strains have been reported.

## ANNOUNCEMENT

Bovine anaplasmosis is an arthropod-borne hemolytic disease caused by Anaplasma marginale (order *Rickettsiales*, family *Anaplasmataceae*), an obligate intraerythrocytic bacterium (1). Bovine anaplasmosis is endemic in Mexico and Central and South America and enzootic in most Latin American countries (2). Bovine anaplasmosis causes important economic losses, mainly due to high morbidity and mortality in cattle (3, 4). The genetic diversity of A. marginale has been reported in several countries, including Mexico (5–10).

Here, we report the draft genome of A. marginale strain Mex-01-001-01, isolated from blood of sick cattle from Aguascalientes, Mexico.

We used 200 µl of bovine blood to extract genomic DNA using the UltraClean DNA BloodSpin kit (Mo Bio Laboratories). Two micrograms of genomic DNA was sequenced with the MiSeq system (Illumina) (Genomics Core of Arizona State University). We obtained a random data set of 1,100,300 paired-end reads of 300 bp which were reported in the SRA database.

The Illumina adapter sequences were removed from Illumina paired-end reads using the ILLUMINACLIP trimming step of Trimmomatic version 0.36, using the default settings (11). Low-quality bases were removed using the dynamictrim algorithm of SolexaQA++ version 3.1.7.1 (12), with a Phred quality score Q of 13. The resulting paired-end reads were *de novo* assembled using SPAdes version 3.11.1 (13) using the options (i) only runs assembly module (–only-assembler), (ii) reduce the number of mismatches (–careful), and (iii) k-mer lengths between 21 and 127. The contigs of A. marginale Mex-01-001-01 were differentiated from those contigs that belong to other organisms (i.e., contigs of bovine genomes) based on the GC content of each A. marginale contig using a Python script (https://github.com/FernandoMtzMx/GC_content_MultiFasta) (the A. marginale genomes reported have GC contents of 46% to 52%). Also, we aligned the sequences of each contig with the nr/nt database using BLASTN (14). Those contigs with an alignment coverage higher than 50% and identity higher than 70% corresponded to A. marginale.

The features of the draft genome were evaluated with QUAST version 4.6.2 using default settings (15).

The draft genome of A. marginale Mex-01-001-01 has 34 contigs with a total length of 1,179,425 bp and an *N*_50_ contig length of 65,428 bp, a GC content of 49.79%, and ∼36× coverage. The alignment of the two FASTQ files and the draft genome of A. marginale Mex-01-001-01 using Bowtie version 2.3.4 (16) revealed that 142,974 paired-end reads belong to A. marginale Mex-01-001-01.

The draft genome sequence of A. marginale Mex-01-001-01 was annotated automatically with the RAST server version 2.0 (17), identifying 1,218 genes and 1,178 coding sequences (CDS). The 16S rRNA gene sequence was obtained using RNAmmer version 1.2 (18), with a length of 1,491 bp, 100% alignment coverage, and 99% and 100% identities with those of A. marginale Florida and A. marginale St. Maries, respectively (GenBank accession number CP001079 and CP000030).

### Data availability.

This whole-genome shotgun project has been deposited at DDBJ/ENA/GenBank under the accession number QLIV00000000. The version described in this paper is QLIV01000000. The Sequence Read Archive (SRA) accession number is SRP157906.
